# Supplementary material for: Sex bias in iron sequestration by transferrin 1 modulates sexually dimorphic infection outcomes in Drosophila melanogaster
Source: Genetics. 2026 Mar 2;233(1):iyag058. doi: 10.1093/genetics/iyag058 (PMC13147530; doi:10.1093/genetics/iyag058)
Supplement: iyag058_Supplementary_Data [file iyag058_supplementary_data.zip › Supplemental_Material_legends_GENETICS-2026-308984.docx]

**Supplemental Material legends**

**Figure S1. The sexually dimorphic survival is independent of the wild-type background and the pathogen dose used for infection. (a-f)** Cumulative survival of mated *Oregon R* flies infected with *P. alcalifaciens* OD = 2 **(a)** and mated *w1118 iso* flies infected with different ODs as indicated **(b-e),** and *PPO1^∆^,2^∆^*  *iso* flies infected with *P. alcalifaciens* OD = 2 **(f)**. n indicates the total number of flies used in the experiments per genotype.

**Figure S2. *Oregon R* flies show sexual dimorphism in *Tsf1* expression and iron hemolymph levels. (a)** RT-qPCR measuring *Tsf1* expression in unchallenged (UC) or flies infected with *P. alcalifaciens* 6 and 16 hours post infection, respectively. One dot represents expression levels from 10 pooled flies. The mean and SD of three independent experiments are shown. Data was analyzed using Two-way ANOVA, Šídák's multiple comparisons test. **(b)** Relative iron levels in the hemolymph of unchallenged (UC) flies. The iron content was measured using the ferrozine assay. One dot represents iron levels of a pool of 75+ flies. Data shown as relative values compared to iron levels in male flies. The mean and SD of three independent experiments are shown. Data was analyzed using a One Sample t-test.

**Figure S3. *Tsf1 iso* flies do not show sexually dimorphic survival irrespective of the initial dose of infection**. **(a-c)** Cumulative survival of *Tsf1 iso* flies infected with different ODs of *P. alcalifaciens* as indicated. n indicates the total number of flies used in the experiments per sex.

**Supplementary tables S1-S6**

**Table S1.** Data used to generate the graphs in Figure 1.

**Table S2.** Data used to generate the graphs in Figure 2.

**Table S3.** Data used to generate the graphs in Figure 3.

**Table S4.** Data used to generate the graphs in Figure 4.

**Table S5.** Data used to generate the graphs in Figure S1.

**Table S6.** Data used to generate the graphs in Figure S2.
