# Supplementary figures and images for: Sex bias in iron sequestration by transferrin 1 modulates sexually dimorphic infection outcomes in Drosophila melanogaster
Source: Genetics. 2026 Mar 2;233(1):iyag058. doi: 10.1093/genetics/iyag058 (PMC13147530; doi:10.1093/genetics/iyag058)

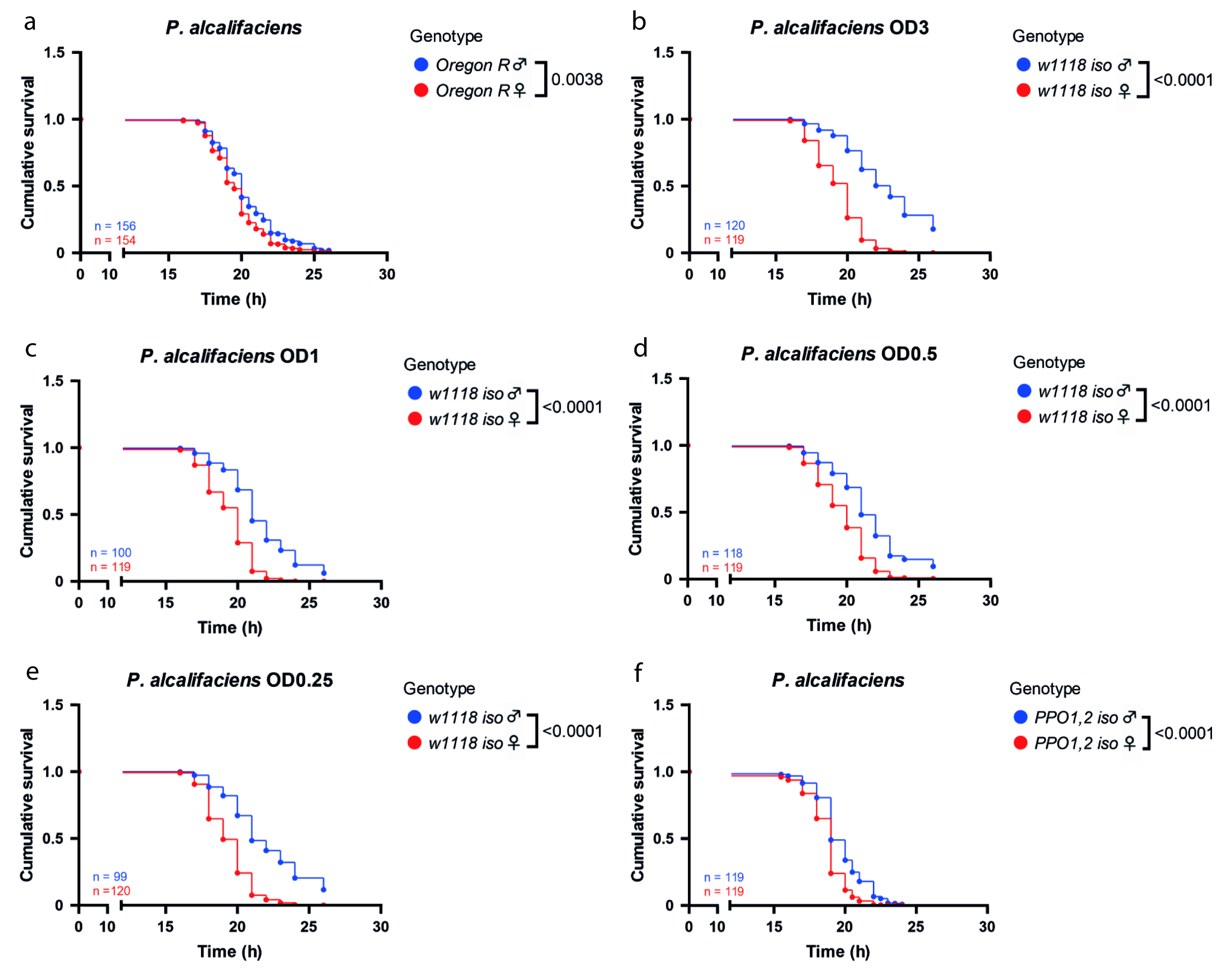

Supplement: iyag058_Supplementary_Data [file iyag058_supplementary_data.zip › Supplemental_Figure_1_GENETICS-2026-308984.tif]

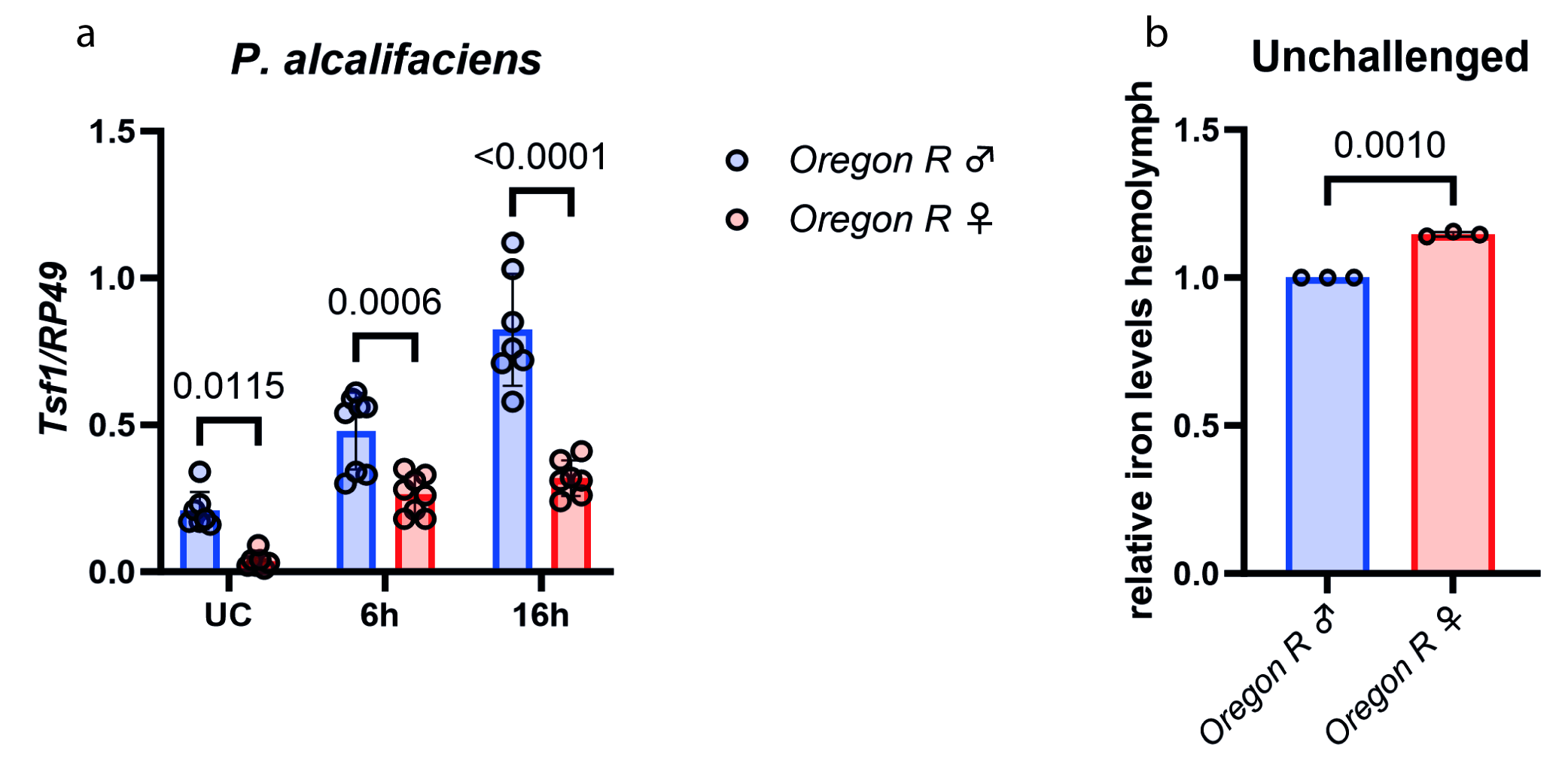

Supplement: iyag058_Supplementary_Data [file iyag058_supplementary_data.zip › Supplemental_Figure_2_GENETICS-2026-308984.tif]

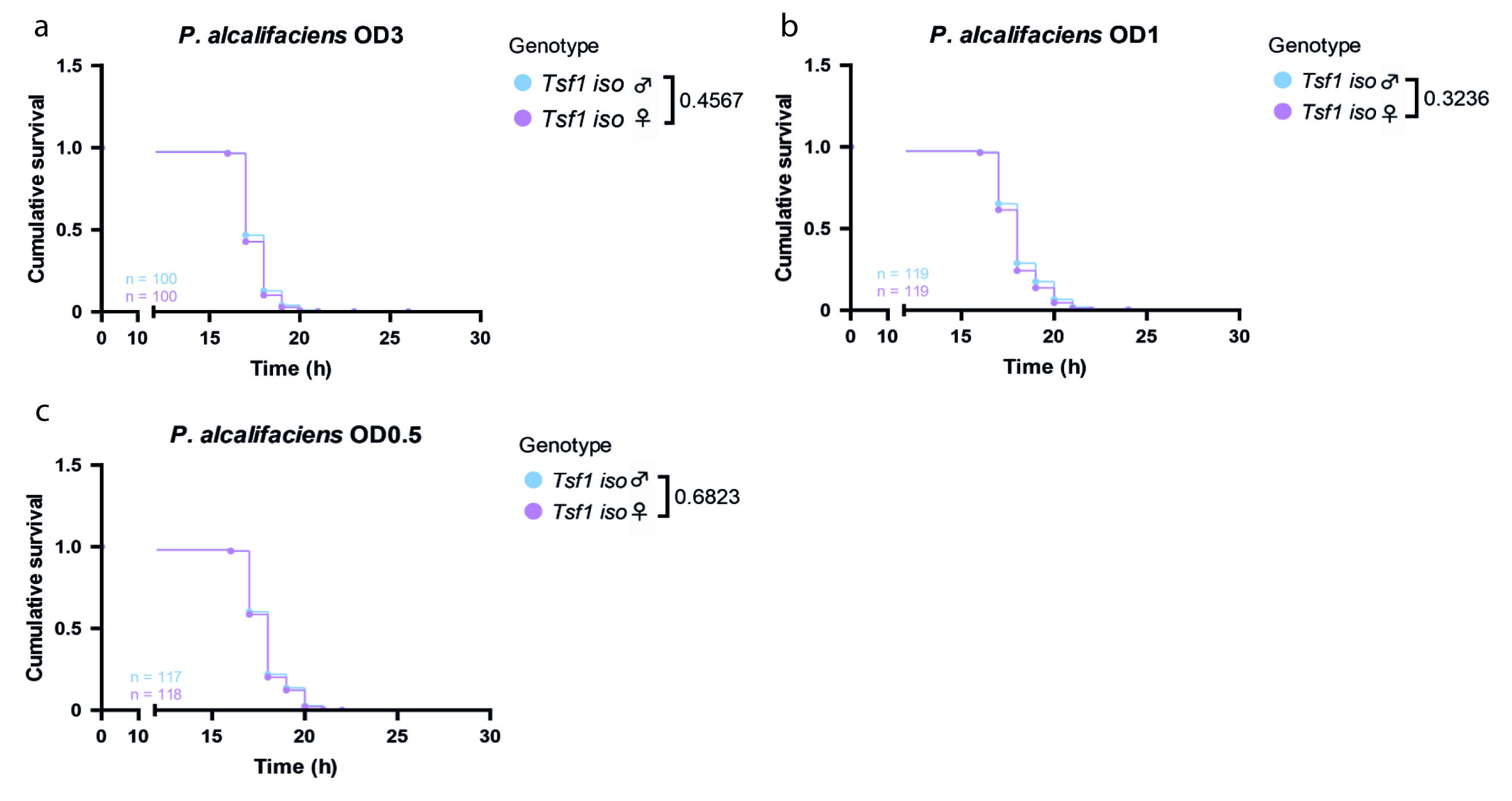

Supplement: iyag058_Supplementary_Data [file iyag058_supplementary_data.zip › Supplemental_Figure_3_GENETICS-2026-308984.tif]
